# Exopolysaccharides-Mediated ZnO Nanoparticles for the Treatment of Aquatic Diseases in Freshwater Fish *Oreochromis mossambicus*

**DOI:** 10.3390/toxics11040313

**Published:** 2023-03-27

**Authors:** Muthukumar Abinaya, Sathappan Shanthi, Jesudasan Palmy, Khalid A. Al-Ghanim, Marimuthu Govindarajan, Baskaralingam Vaseeharan

**Affiliations:** 1 Biomaterials and Biotechnology in Animal Health Lab, Department of Animal Health and Management, Alagappa University, Science Block, 6th Floor, Burma Colony, Karaikudi 630004, Tamil Nadu, India; govindamalmuthukumar06@gmail.com (M.A.); shanthisathappan@gmail.com (S.S.); 2 Poultry Production and Product Safety Research Unit, ARS, USDA, Center of Excellence for Poultry Science, University of Arkansas, 1260 W Maple St., Fayetteville, AR 72701, USA; palmy.jesudhasan@usda.gov; 3 Department of Zoology, College of Science, King Saud University, Riyadh 11451, Saudi Arabia; kghanim@ksu.edu.sa; 4 Unit of Vector Control, Phytochemistry and Nanotechnology, Department of Zoology, Annamalai University, Annamalainagar 608002, Tamil Nadu, India; drgovind1979@gmail.com; 5 Department of Zoology, Government College for Women (Autonomous), Kumbakonam 612001, Tamil Nadu, India

**Keywords:** *Bacillus licheniformis*, zinc nanoparticle, immune parameters, antioxidant response, growth performance, aquatic pathogens

## Abstract

Bacterial fish disease outbreaks are a key concern for aquaculture. Complementary feed additives such as immunostimulants can serve as an ideal solution for disease prevention. Herein, we scrutinized the efficacy of exopolysaccharides (EPSs) from probiotic *Bacillus licheniformis* and EPS-mediated zinc oxide nanoparticles (EPS-ZnO NPs) for a diet to evaluate growth parameters, antioxidant enzyme activities, and immune stimulation together with disease resistance against *Aeromonas hydrophila* and *Vibrio parahaemolyticus* in Mozambique tilapia *Oreochromis mossambicus*. Fish were separated into seven groups, with six experimental groups fed with EPS and EPS-ZnO NPs at 2, 5, and 10 mg/g and a control fed a basal diet. The fish ingesting feed supplemented with EPS and EPS-ZnO NPs at 10 mg/g showed improved growth performance. Cellular and humoral-immunological parameters were tested in serum and mucus after 15 and 30 days of feeding. These parameters were substantially enriched with a 10 mg/g diet (*p* < 0.05) of EPS and EPS-ZnO NPs in comparison with the control. Furthermore, the EPS and EPS-ZnO NP supplemental diet actively enhanced the antioxidant response (glutathione peroxidase, superoxide dismutase, and catalase). In addition, the supplemental diet of EPS and EPS-ZnO NPs lowered the death rate and improved the disease resistance of *O. mossambicus* following assessment with *A. hydrophila* and *V. parahaemolyticus* at 50 µL. Hence, the overall results suggest that the supplemental diet of EPS and EPS-ZnO NPs might be used to ensure aquaculture feed additives.

## 1. Introduction

Aquaculture is the most important agriculture industry, and it has grown far faster than other food sectors to satisfy fish market requirements. Around 15% of the protein ingested by three billion people comes from fish. In emerging nations such as South Asia, Southeast Asia, China, and Japan, fish is considered a primary nutrition source, creating a massive demand for intensive farming. With the expansion of industry and increased yields, intensive culture practices have been adopted that lead to environmental stress and disease prevalence, which are considered essential challenges for the sector [1,2,3,4]. Bacterial infections are the main issue in Indian aquaculture. *Vibrio* spp. and *Aeromonas hydrophila* are the most prevalent and harmful bacterial pathogens that challenge survival rates in both commercially important marine and freshwater fish [5,6,7,8,9,10].

The utilization of antibiotics and chemotherapeutics to suppress infections in fish is commonly criticized for its adverse impacts. The excess use of these agents has brought up a few concerns, such as antibiotic resistance and the appearance of residue in tissues and the environment. Likewise, many traditional ways of preventing infectious illnesses and improving fish growth parameters in aquaculture have been explored [11,12,13,14].

Aquaculture applications pay much attention to natural products that build up the immune systems of fish in order to enrich their growth and resilience to infection [15]. In aquaculture, some immune-stimulant plants, prebiotics, and probiotics can serve as ideal alternative feed additives for disease prevention without any hazardous effects on fish, human health, or the environment [16,17,18,19].

Nanotechnology is well thought out as a newer way to synthesize affordable materials for the remediation of water bodies [20]. Mainly, the level of zinc in a diet may undesirably deteriorate the condition of an additional element such as Cu (copper), Fe (iron), or Cd (cadmium) [21]. It increases the feed cost and subsidizes the minerals in the marine environment [22]. Consequently, the usage of trace minerals at the nano-dimension is a creative solution, which has led to fewer quantities being used to promote growth and immunity against the influence of antioxidants [23,24]. Likewise, nano-sized minerals have catalytic proficiency, more robust adsorbing capacity, enriched bactericidal ability, and greater bioavailability when compared with normal-sized particles [25,26].

In recent decades, numerous polysaccharides have been identified as biological response modifiers to potent immunostimulatory factors and used as biomaterials to prepare eco-friendly nanoparticles (NPs). Compared with polymers from other sources, microbial EPS can be formulated under precise constraints with enduring stability in the GI tract to enrich the colonization of useful microorganisms. They can also effectively act as a strong reducing and stabilizing agent for the production of metal NPs. Due to their properties and structures with many functional groups, EPSs are ideal and efficient for producing metal NPs destined to be applied in the biomedical sciences. Even though EPSs are initiated by macrophages and have a host-mediated anticancer effect, they have remarkable immunomodulatory capabilities [27]. However, limited information can be found on the impact of EPSs on bioactivities, e.g., fish immunomodulation [28]. In this study, we examined the anti-infective potential of exopolysaccharide-mediated zinc nanoparticles against *A. hydrophila* and *V. parahaemolyticus* using *O. mossambicus*.

## 2. Materials and Methods

### 2.1. Experimental Animal

*Oreochromis mossambicus* (average weight: 7.7 ± 1.2 g and length: 8 ± 0.5 cm) was bought from Victoria fish farm and aquarium in Karaikudi, Tamil Nadu, India and was acclimatized for 2 weeks before the feeding trial. According to the method of Gobi et al. [29], fish were sustained in a 300 L FRP tank and provided with a commercial feed (ad libitum; Tairoun Feed Company, Taipei, Taiwan) twice/day at 2% of their body weight. Water temperature was sustained at 29 °C, pH at 6.91 ± 1.44, salinity at 0.25 ± 0.05 ppt, and DO at 6.63 ± 7.78 mg^−1^.

### 2.2. Methods for EPS Extraction and EPS-ZnO NP Synthesis

We extracted exopolysaccharides (EPS) from *B. licheniformis* Dahb1 using ice-cold ethyl alcohol precipitation [30]. Inoculated *B. licheniformis* Dahb1 cultures were fermented at 37 °C for 72 h in 500 mL of nutrient broth medium. After 10 min at 8000 g, the enzymes were inactivated, and the cells were collected. Adding three liters of cold 95% ethanol to the supernatant solution containing EPS and cooling it overnight precipitated the EPS (20 °C). EPS was precipitated after 10 min of centrifuging at 8000× *g*, washing twice with distilled water, and storing in a desiccator. The crude EPS powder was utilized to make ZnONPs. Coprecipitation was used to biosynthesize EPS-mediated zinc oxide nanoparticles (EPS-ZnO NPs) [31]. An amount of 0.5 g of EPS crude powder was added to an aqueous solution (0.02 M) of zinc acetate and NaOH (2 M) at 37 °C while stirring quickly. The white material separated after 10 min of centrifuging at 8000 g and was washed thrice with clean water and 100% ethanol. Before further examination, the precipitate was washed and dried at 70 °C.

### 2.3. Experimental Diet Formulation

In the diet formulation, powdered samples were used for experimental purposes. The commercial diet was blended with EPS and EPS-ZnO NPs dissolved in distilled water (D.H_2_O) at the desired concentrations (control (0), 2, 5, and 10 mg/g). The diet pellets were left to dry before being stored at 4 °C [17,32].

### 2.4. Experimental Design and Sample Collection

At the pretest, fish were selected randomly and split into seven groups with three replicate tanks. Each glass tank (300 L) contained 30 fish with aeration-filtration, and water parameters (temperature, DO, pH, and photoperiod) were scrutinized daily. Each treatment group was fed a different concentration of EPS and EPS-ZnO NPs (control, 2, 5, and 10 mg/g). Fish were fed twice a day for 30 days. The tanks were cleaned biweekly. At 15 and 30 days, five fish were selected randomly from each tank in triplicate to measure the growth performance. After measuring growth parameters, liver tissue, blood serum, and mucus were collected according to the guidelines for the Use of Fish in Research to analyze non-specific cellular and humoral immune parameters and antioxidant enzyme activities [33].

#### 2.4.1. Liver Tissue Collection

The total weight of 5 fish livers (1.0 g) was pulverized into a buffer (10 mL) containing sodium phosphate (25 mM; pH 7.4); protease inhibitor (0.1 mM); dithiothreitol (DTT; 0.1 mM); EDTA (1.0 mM); and phenylthiourea (0.1 mM). Afterward, it was centrifuged at 30 min for 10,000× *g*. The supernatants were excluded and kept at −20 °C for further investigation.

#### 2.4.2. Blood and Serum Collection

Two mL of fish blood was drawn through a caudal puncture in which ≈one mL of blood was imported to a heparinized vacutainer tube and centrifuged at 10 min for 5000× *g*. Later, the excluded serum was retained at −20 °C for further investigation.

#### 2.4.3. Skin Mucus Collection

Each fish was imported into a polyethylene bag containing NaCl (10 mL; 50 Mm) and gently shaken for 2 min. Then, to collect the pooled mucus, the bag was centrifuged at 10 min for 1500× *g*. Afterward, the supernatant was filtered (0.45 µm) and retained for later use at −20 °C.

### 2.5. Growth Performance

After 30 days of the experimental diet, growth rates and feed intake were arbitrated as follows:

Weight gain (WG; g/fish) = W_t_ − W_0_

Specific growth rate (SGR) = 100 × [(W_t_ −W_0_)/t]

Feed conversion ratio (FCR) = FI/(W_t_ − W_0_)

where W_t_ is the final weight; W_0_ is the initial weight; t is the interval of feeding, and FI is the feed ingestion.

### 2.6. Immune Response

#### 2.6.1. Cellular-Immunological Parameters

##### Myeloperoxidase (MPO) Activity

With minor changes, the MPO action of the fish serum and mucus was determined as per the technique illustrated by Kumari and Sahoo [34]. In brief, 20 μL sample (serum and mucus) was diluted with HBSS, and 50 μL freshly prepared myeloperoxidase substrate (5 mM of hydrogen peroxide and 20 mM of 3, 3′, 5, 5′- tetramethylbenzidine hydrochloride) was placed for 30 min at 30 °C. Thenceforth, the reaction was ended by including 35 μL sulphuric acid (4 M), and OD_450_ nm was inferred with an ELISA plate.

##### Reactive Oxygen Species (ROS) Activity

The ROS activity was investigated with the technique illustrated by Secombes [35]. In short, fish blood regulated with an anticoagulant (heparin) solution was loaded into ninety-six-well plates and incubated for 30 °C at 60 min. After incubation, PBS-pH 7.2 was used to rinse the wells to eradicate non-adhered cells. Afterward, 50 μL of NBT was added to incubate at the same interval. For the cell fixation, 30% of CH_3_OH was added and left to settle for three minutes. Lastly, potassium hydroxide (60 μL; 2 M) plus DMSO (70 μL) was filled in each well, and absorbance was read at OD_540_ nm.

##### Reactive Nitrogen Species (RNS) Activity

For RNS activity, Griess reagent was used to determine the fish serum nitric oxide (NO) level [36]. Briefly, 2 mL of 10 mM sodium-nitroprusside was liquefied with PBS (0.5 mL; pH 7.4); to that, serum (100 μL) was added prior to incubation at 25 °C. After 2 h, the Griess reagent (0.5 mL) was added and left at 37 °C for 30 min to infer the absorbance at OD_546_ nm.

#### 2.6.2. Humoral-Immunological Parameters

##### Antiprotease Activity

The serum antiprotease assay was performed as described by Bowden et al. [37] with subtle changes. Concisely, serum (10 μL) was added among 20 μL trypsin, followed by BAPNA substrate (500 μL), and the concentration was made again with Tris-HCl (1 mL; 0.1 M). After incubation for 25 min at 22 °C, the action was ended by including acetic acid (30%; 150 μL), and the proportion of trypsin inhibition was measured at OD_415_ nm.

##### Lysozyme-LYZ Activity

Lysozyme activity of the fish serum and mucus was assessed using Gram+ *Micrococcus lysodeikticus* bacteria (Sigma USA) [38]. In this, *M. lysodeikticus* (1 ml; 0.2 mg/mL) was kept in PBS (pH:6.2; 0.05 M). To this, 100 μL of the sample (serum and mucus) was added and left for 1 h to interpret the absorbance at OD_530_ nm.

##### Alkaline Phosphatase-ALP Activity

The alkaline phosphatase assay was performed with the following procedure of Sanchooli et al. [39]. Concisely, the serum and mucus (50 μL) were incubated with NH_4_HCO_3_ (150 μL; pH: 7.8; and 100 mM) containing MgCl_2_ (1 mM) for 15 min at 30 °C. Afterward, 50 μL of 4 mM p-nitrophenyl phosphate (substrate) was mixed, and the absorbance was interpreted at OD_405_ nm.

##### Hemolytic Complement Activity

The hemolytic complement ability of the fish serum was evaluated using Human RBC as elucidated by Cuesta et al. [40]. For this assay, serum was diluted 2-fold with 100 μL of HBSS containing 1 mM Mg^2+^, 10 mM EGTA, and 6.7 mM of HEPES. Afterward, 100 μL HuRBCs was added and left at 22 °C for 90 min. The reaction was then terminated using 1 mL HBSS containing 10 mM EDTA. Finally, a centrifuge of the non-lysed HuRBCs (500× *g*; 5 min) and the supernatant were used to read the activity at OD_405_ nm.

### 2.7. Antioxidant Enzymes Activity

#### 2.7.1. Superoxide Dismutase Activity

This assay was completed with the spectrophotometric technique clarified by Suzuki et al. [41] with slight changes. Briefly, O_2_^−^ producer (xanthine oxidase; 0.1 μM/mL in 2 M (NH₄)₂SO₄), 50 mM sodium carbonate (xanthine: 0.1 mM; NBT-indicator: 0.025 mM and EDTA: 0.1 mM), and serum/blank-PBS were assorted and calculated at OD_560_ nm.

#### 2.7.2. Catalase Activity

The catalase assay was performed using the procedure of Takahara et al. [42] with subtle changes. In brief, serum (100 μL) was dispersed with phosphate buffer (1.2 mL), followed by 1 mL of hydrogen peroxide suspension. After 5 min, the absorbance diminution was read at OD_240_ nm.

#### 2.7.3. Glutathione Peroxidase Activity

The protocol of Rotruck et al. [43] was modified to determine the GPx activity. Briefly, a mixture of 0.1 mL serum, 0.1 mL H_2_O_2_, 0.2 mL EDTA, 0.1 mL sodium azide, 0.4 mL phosphate buffer, and 0.2 mL GSH was incubated for 10 min at 37 °C. To detain the response, trichloroacetic acid (TCA; 0.5 mL) was added and centrifuged at 2000× *g*. Afterward, 1 mL of 5,5′-dithiobis (2-nitrobenzoic acid DTNB) with 3 mL of Na_2_HPO_4_ were added on to the obtained supernatant and read at OD_420_ nm.

### 2.8. Challenge Test with A. hydrophila and V. parahaemolyticus

This investigation explored the possibility of using EPS and EPS-ZnO NPS-supplement diets to combat against *A. hydrophila* and *V. parahaemolyticus* infection in *O. mossambicus*. Ten fish were injected intraperitoneally from all groups with an *A. hydrophila* and *V. parahaemolyticus* bacterial cell suspension (50 μL; 1 × 10^7^ cells/mL). The tested fish were scrutinized daily for 2 weeks to document mortality [44].

### 2.9. Statistical Analyses

A statistical study was performed by applying 1-way ANOVA with SPSS version 16.0. Tukey’s multiple comparisons post hoc was applied to relate the means of different groups, where the statistical implication was accepted at *p* < 0.05. The outcomes were displayed as means ± SD.

## 3. Results

### 3.1. Growth Performance

Table 1 shows the impact of the EPS and EPS-ZnO NPs diet on the body mass of *O. mossambicus*. After a 30-days trial in *O. mossambicus*, appreciable mass differences in growth rates were noticed when compared with the control (without diet). However, compared to EPS, the highest effect on Wt, SGR, and FCR was noticed at 10 mg/g of EPS-ZnO NPs fed to fish. In the control, the diet failed to prompt any substantial growth parameters.

### 3.2. Immune Response

#### 3.2.1. Cellular Immunological Parameters

##### Myeloperoxidase-MOP Activity

**Figure 1:**
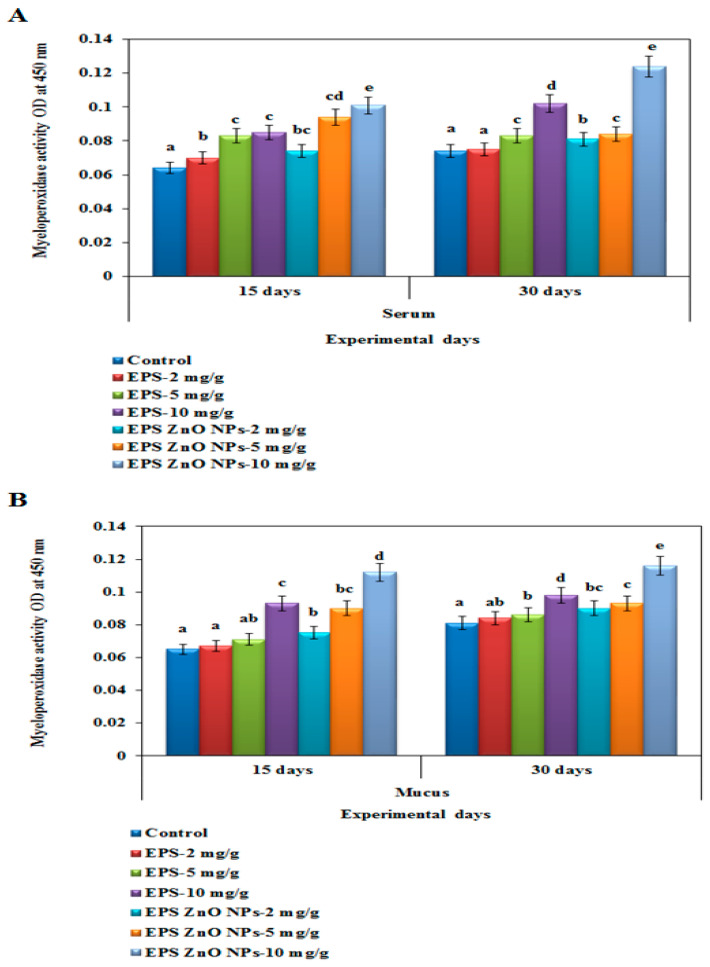
Myeloperoxidase activity of *O. mossambicus* fed with EPS and EPS-ZnO NPs: (**A**) serum and (**B**) mucus. T-bars indicate three replicates of mean ± SD, and different letters correspond to the statistical difference between treated groups.

After thirty days of the trial diet with EPS and EPS-ZnO NPs, serum and mucus MOP activity were elevated significantly more than in the control (Figure 1A,B). In fish serum on day 15, the fish fed with EPS and EPS-ZnO NPs (10 mg/g) showed 0.085 U and 0.101 U, whereas on day 30, the MOP level increased to 0.102 U and 0.124 U, respectively. In fish mucus on day 15, fish fed with EPS and EPS-ZnO NPs (10 mg/g) showed 0.093 U and 0.112 U, whereas on day 30, the MOP level increased to 0.098 U and 0.116 U, respectively. Our findings show that a diet containing EPS-ZnO NPs was more potent than EPS in enhancing the MOP activity of mucus and was more significant in both diet groups than the serum of treated fish. In comparison, the control groups showed no improvement.

##### ROS and RNS Activity

**Figure 2:**
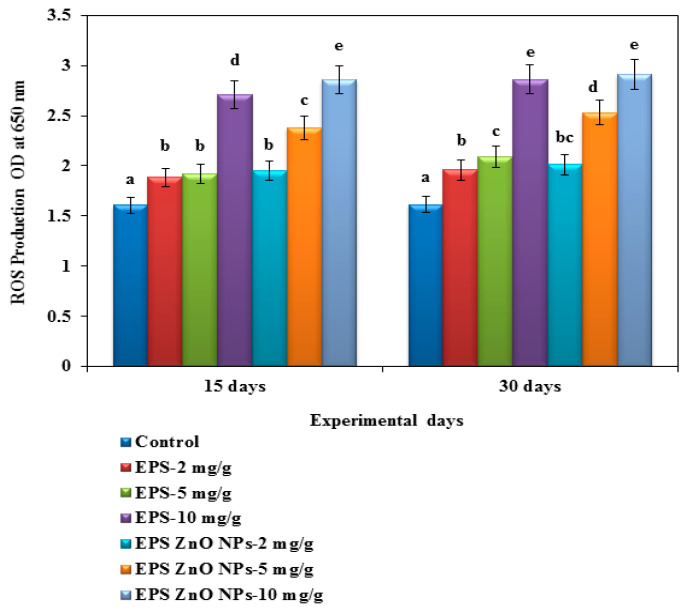
Respiratory burst activity/reactive oxygen species production/nitroblue tetrazolium (NBT) assay of *O. mossambicus* fed with EPS and EPS-ZnO NPs. T-bars indicate three replicates of mean ± SD, and different letters correspond to the statistical difference between treated groups.

**Figure 3:**
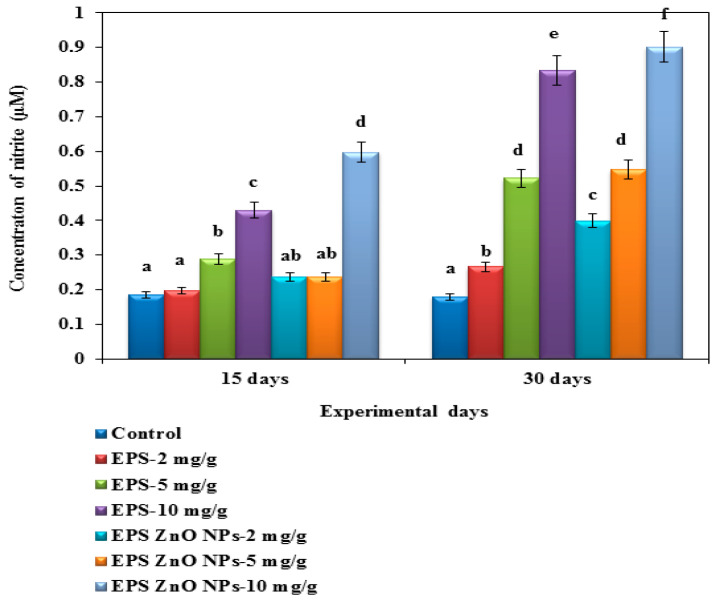
Reactive nitrogen species of *O. mossambicus* fed with EPS and EPS-ZnO NPs. T-bars indicate three replicates of mean ± SD, and different letters correspond to the statistical difference between treated groups.

Figure 2 and Figure 3 present the efficacy of the EPS and EPS-ZnO NPs diet on ROS and RNS activity. On day 30, fish fed with 2, 5, and 10 mg/g of EPS- ZnO NPs showed increased ROS activity at 2.013 U, 2.532 U, and 2.912 U, and those on the EPS diet showed 1.614 U, 1.959 U, and 2.093 U, respectively. Conversely, 10 mg/g of the EPS-ZnO NPs diet showed greater RNS activity at 0.902 U, whereas it was significantly lower in the group fed with 10 mg/g of EPS at 0.834 U. A significant difference was not noticeable in the control group.

#### 3.2.2. Humoral Immunological Parameters

##### Antiprotease Activity

**Figure 4:**
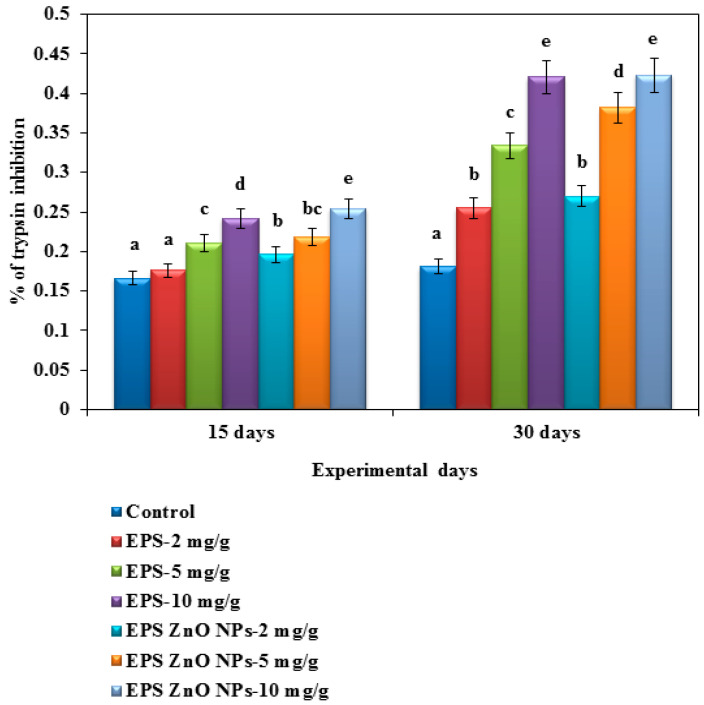
Antiprotease activity of *O. mossambicus* fed with EPS and EPS-ZnO NPs. T-bars indicate three replicates of mean ± SD, and different letters correspond to the statistical difference between treated groups.

In this assay, the effect of different concentrations of the EPS and EPS-ZnO NP-supplemented diet (2, 5, and 10 mg/g) on the serum antiprotease of experimental fish was illustrated on day 15 and 30 (Figure 4). For day 30, fish fed with EPS-ZnO NPs were elevated at 0.270, 0.382, and 0.423 U, whereas those fed with EPS were 0.255, 0.334, and 0.421 U, respectively. However, fish fed with both diets had intensified serum antiprotease action levels, and a significant difference was not noticeable in the control.

##### Lysozyme-LYZ Activity

**Figure 5:**
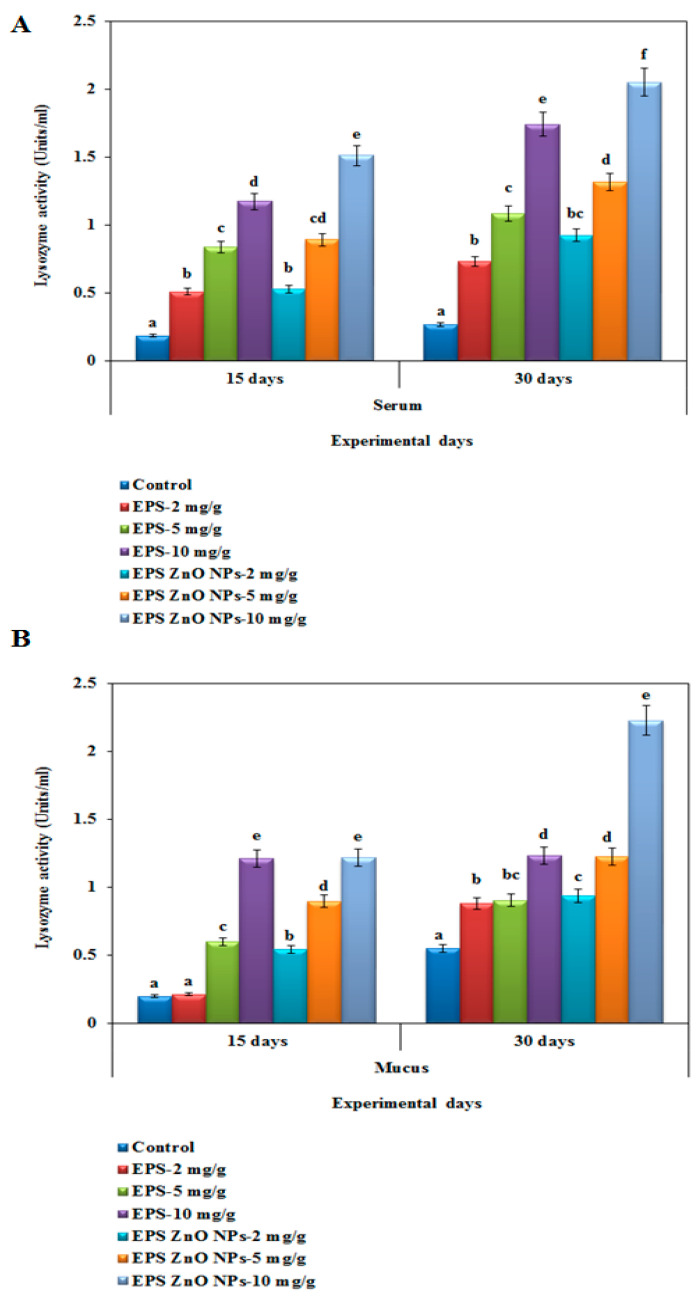
Lysozyme activity of *O. mossambicus* fed with EPS and EPS-ZnO NPs: (**A**) serum and (**B**) mucus. T-bars indicate three replicates of mean ± SD, and different letters correspond to the statistical difference between treated groups.

In this assay, the mucus and serum of LYZ activity were increased substantially (*p* < 0.05) in the EPS and EPS-ZnO NPs experimental diet groups and compared to control groups after 15 and 30 days of the feeding trial (Figure 5A,B). In fish serum on day 15, those fed EPS and EPS-ZnO NPs (10 mg/g) showed 1.173 U and 1.511 U, whereas on day 30, lysozyme activity was increased to 1.74 U and 2.049 U, respectively. In fish mucus on day 15, those fed with EPS and EPS-ZnO NPs (10 mg/g) showed 1.21 U and 1.217 U, whereas on day 30, the level of myeloperoxidase was increased to 1.23 U and 2.227 U, respectively. The mucus showed the highest LYZ level, which correlated with serum after 30 days of the diet.

##### Alkaline Phosphatase-ALP Activity

**Figure 6:**
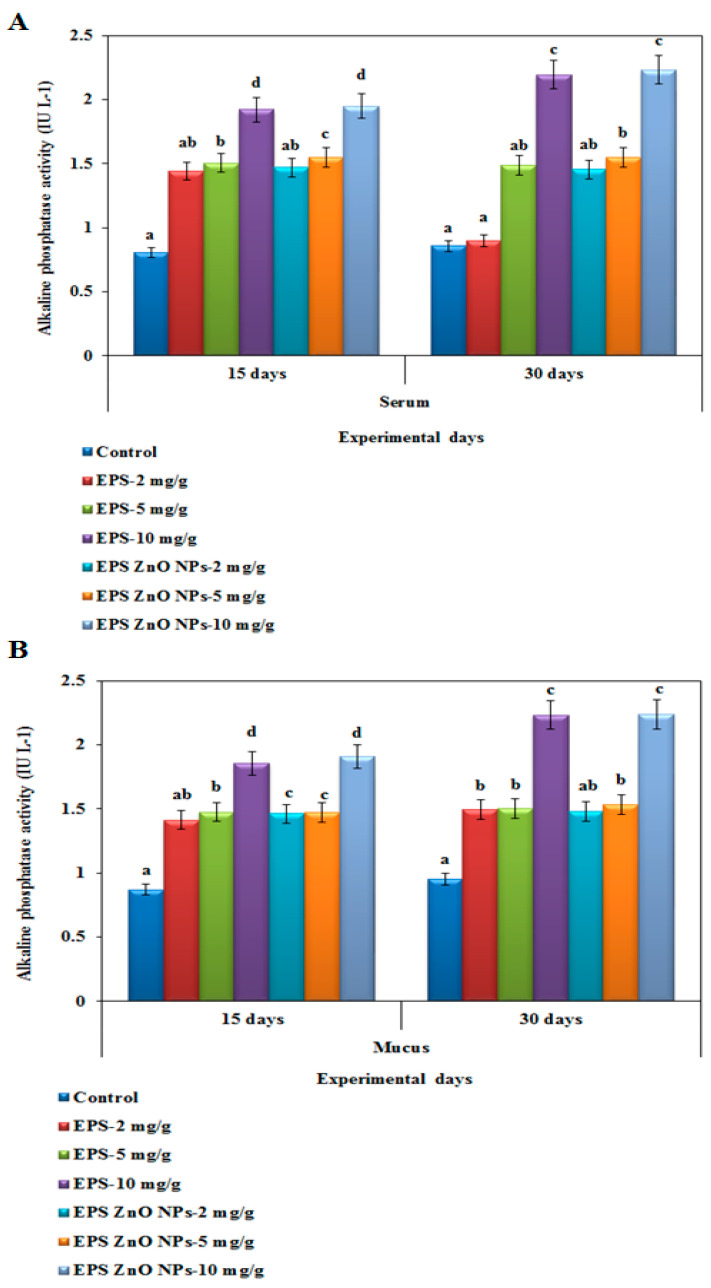
Alkaline phosphatase activity of *O. mossambicus* fed with EPS and EPS-ZnO NPs: (**A**) serum and (**B**) mucus. T-bars indicate three replicates of mean ± SD, and different letters correspond to the statistical difference between treated groups.

Figure 6A,B show the ALP activity of serum and mucus in fish fed with EPS and EPS- ZnO NPs. Higher alkaline phosphatase activity was noted at 10 mg/g of the EPS-ZnO NPs diet (0.421 U), which corresponded to EPS (0.423 U) on day 30. Conversely, lower activity was shown with 2 mg/g of EPS (0.255 U) on day 30.

##### Hemolytic Complement Activity

**Figure 7:**
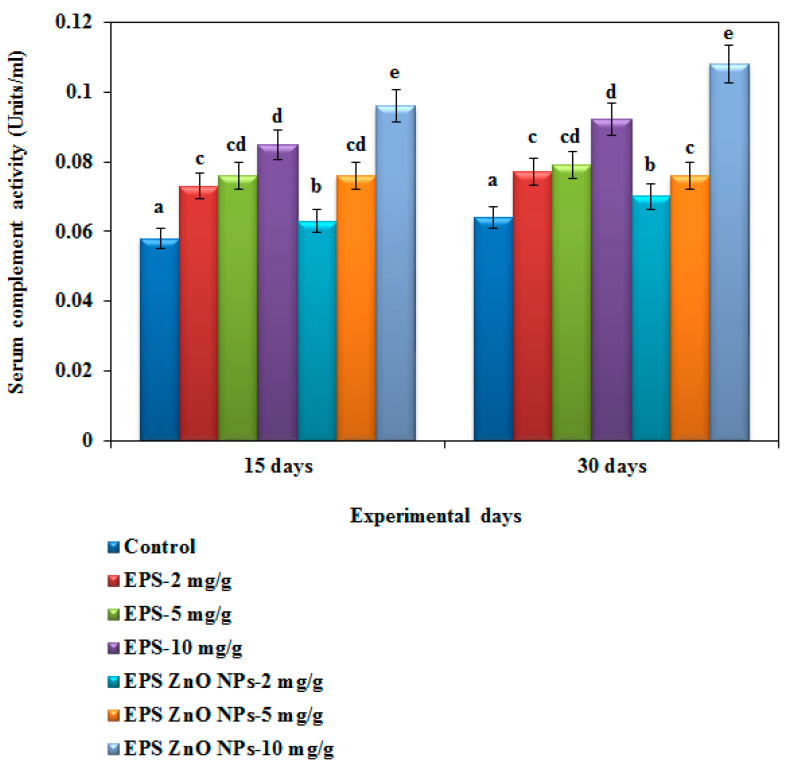
Serum innate immune complement hemolytic activity of *O. mossambicus* fed with EPS and EPS-ZnO NPs. T-bars indicate three replicates of mean ± SD, and different letters corresponding to the statistical difference between treated groups.

The hemolytic complement activity of the serum of fish fed with the EPS and EPS-ZnO NPs dietary supplement is revealed in Figure 7. However, the EPS-ZnO NPs diet showed a drastically elevated complement action of 0.096 U on day 15 and 0.108 U on day 30, in contrast to the EPS diet with 0.085 U on day 15 and 0.092 U on day 30. The control had no intensified complement action (0.058 on day 15 and 0.064 on day 30).

### 3.3. Antioxidant Enzyme Activity

**Figure 8:**
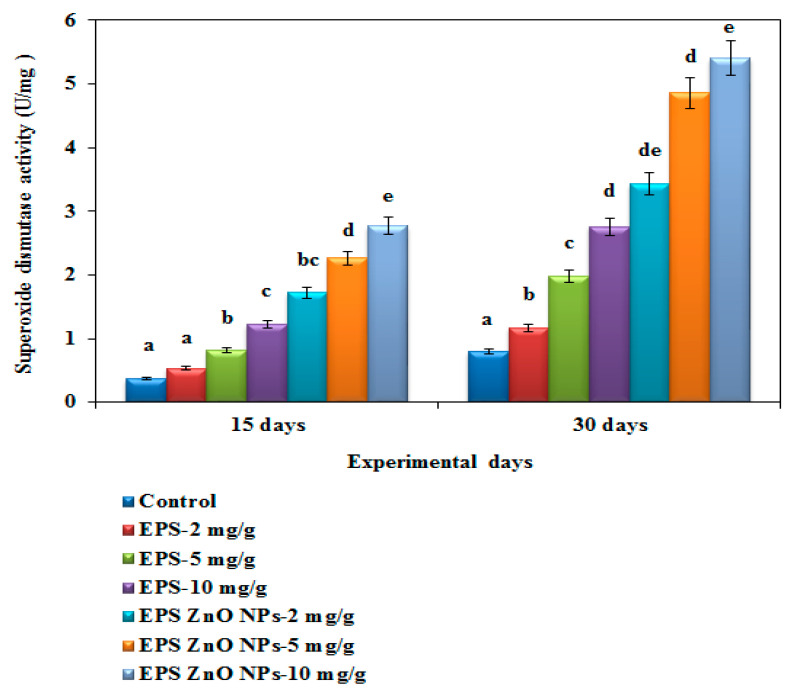
Superoxide dismutase activity (SOD) of *O. mossambicus* fed with EPS and EPS-ZnO NPs. T-bars indicate three replicates of mean ± SD, and different letters correspond to the statistical difference between treated groups.

**Figure 9:**
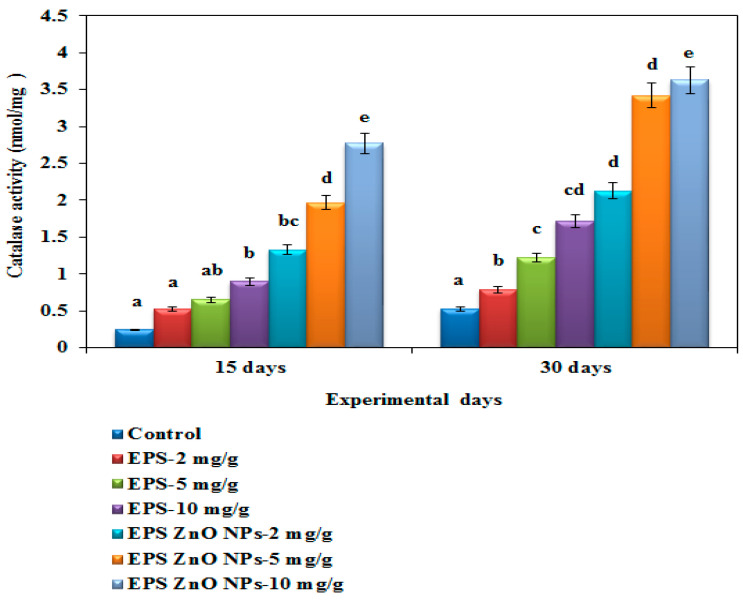
Catalase activity (CAT) of *O. mossambicus* fed with EPS and EPS-ZnO NPs. T-bars indicate three replicates of mean ± SD, and different letters correspond to the statistical difference between treated groups.

**Figure 10:**
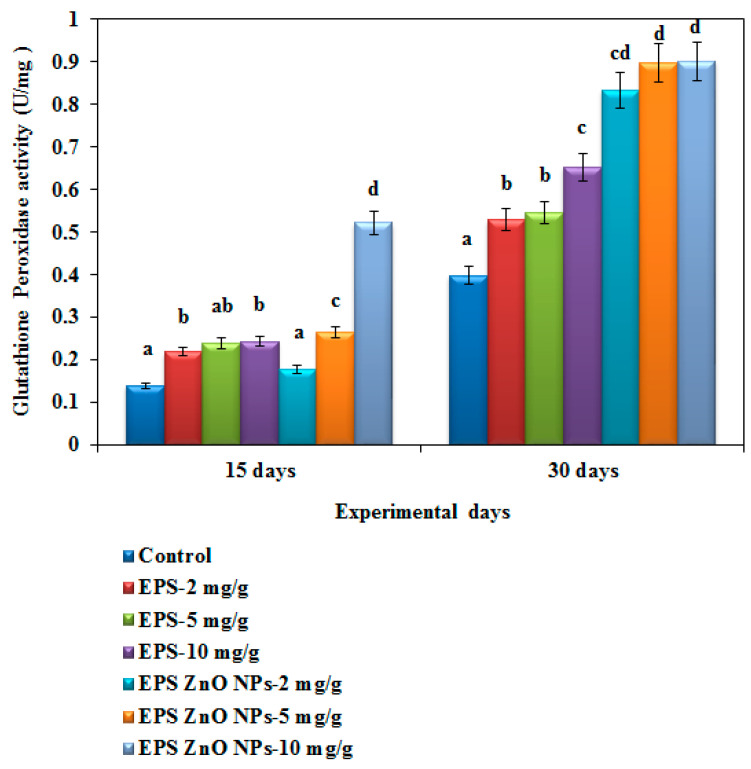
Glutathione peroxidase activity (GPx) of *O. mossambicus* fed with EPS and EPS-ZnO NPs. T-bars indicate three replicates of mean ± SD, and different letters correspond to the statistical difference between treated groups.

The SOD activity of the fish fed with the EPS-ZnO NPs diet was extensively higher, at 0.897 U/mg and 1.72 U/mg on day 15 and 30, respectively, compared to those fed with EPS (2.77 U/mg on day 15 and 3.63 U/mg on day 30) (Figure 8). The catalase activity of fish fed with the EPS and EPS-ZnO NPs diet was drastically higher compared with the control: on day 30, fish fed with 10 mg/g of EPS and EPS-ZnO NPs showed 2.77 U and 5.40 U of catalase activity (Figure 9). In glutathione peroxidase activity, fish fed with the EPS and EPS-ZnO NP-supplemented diet were elevated with the highest dose (10 mg), and the values observed were at 0.52 U and 0.902 U (Figure 10). Finally, SOD, CAT, and GPx were enriched pointedly at *p* < 0.05 in the EPS and EPS-ZnO NPs diet in comparison to the control group.

### 3.4. Challenge Test with A. hydrophila and V. parahaemolyticus

**Figure 11:**
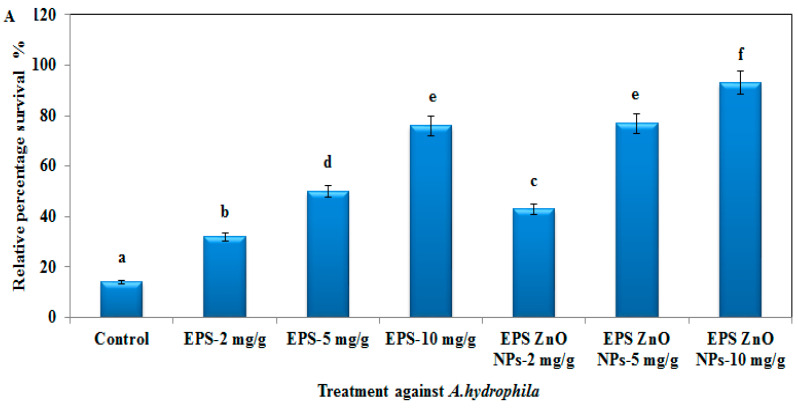

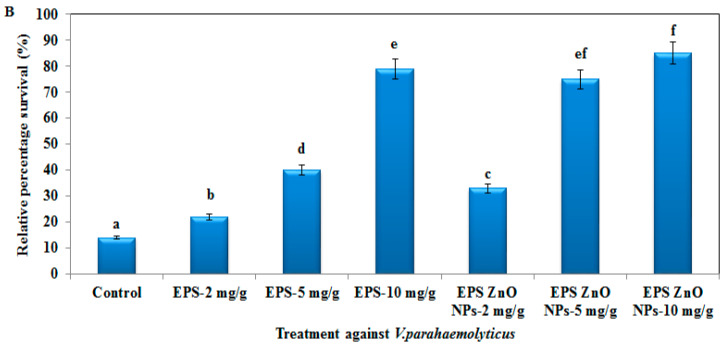
The survival rate of *O. mossambicus* fed with EPS and EPS-ZnO NPs against *A. hydrophila* (**A**) and *V. parahaemolyticus* (**B**). T-bars indicate three replicates of mean ± SD, and different letters correspond to the statistical difference between treated groups.

When challenged with *A. hydrophila* and *V. parahaemolyticus*, fish fed with the EPS and EPS-ZnO NPs diet survived substantially more than the control. Fish fed with the EPS-ZnO NPs diet displayed a higher rate of survival (93% in *A. hydrophila* and 85% in *V. parahaemolyticus*) compared to those fed with EPS (76% in *A. hydrophila* and 79% in *V. parahaemolyticus*) (Figure 11). Compared to the control, *O. mossambicus* fish had lower mortality with the experimental diet.

## 4. Discussion

In the last decade, aquaculture has faced numerous difficulties with the emergence of pathogens and drug consumption limits for disease treatment [45]. Medicinal plants and bioactive compounds have been used for animal health [46,47,48,49,50]. The role of probiotics in enriching fish growth parameters, immune response feed efficiency, and modulating gut microflora was already well known [18,51,52]. Several studies have been published on the proficiency of *Bacillus* spp. [53,54,55,56] to produce extracellular enzymes in the GI (gastrointestinal) tracts of freshwater fish such as *Labeo rohita* [57,58], *Oreochromis niloticus* [59], and *Ephinephelus bruneus* [60]. However, there seems to be no research into the efficacy of dietary microbial polysaccharides on *O. mossambicus*. The anti-infective potential of EPS and EPS-ZnO NPs with regard to the growth performance, immunity, and disease resistance of Mozambique tilapia *O. mossambicus* against aquatic pathogens was investigated in this study.

Regarding growth parameters, our findings revealed that fish fed with EPS and EPS-ZnO NPs had an increased growth rate and noticeable mass differences with an amount of 10 mg/g after 30 days. In the same way, significant improvements in the growth of *Oncorhynchus mykiss* [61] and *Rutilus frisii kutum* [62] fed with probiotic bacteria have been reported.

In fish, cellular-immunological parameters are the foremost line of defense against intruding pathogenic microbes [63]. Our results strongly demonstrate that feeding trials with EPS and EPS-ZnO NPs were substantial at *p* < 0.05 and increased both serum and mucus MOP activity after 15 and 30 days. EPS-ZnO NPs were more effective than EPS in enhancing the MOP activity of mucus, which was greater in both diet groups as shown in the treated fish serum. These results correlated with the reports on increased MOP activity in serum, which includes *Oncorhynchus mykiss* fed *B. subtilis* [64]; *P. hypophthalmus* fed *B. licheniformis* Dahb1 [30]; catla fingerlings fed *B. amyloliquefaciens* [52]; and gilthead seabream fed a dietary mix of *Bacillus* spp. and date palm fruit [65]. The ROS and RNS investigation showed higher production in the fish serum of the group fed with EPS and EPS-ZnO NPs at 10 mg/g. Our results were comparable with Wu et al. [66], who reported enriched ROS and RNS generation in *Sophora flavescens*. Likewise, the dietary feed of *T. cordifolia* [67], *S. trilobatum* [68], and *E.alba* [69] in *O. mossambicus* has been reported.

Humoral-immunological parameters are mediated via serum antibodies, which must be secreted with plasma cells and bind to antigens to eliminate them [70]. The maximum lysozyme activity was seen in the mucus following a 30-day immune-boosting diet, which was correlated with the serum. In fish serum on day 30, those fed with EPS and EPS-ZnO NPs (10 mg/g) showed 1.74 U and 2.049 U, and in fish mucus on day 30, it was increased to 2.3 U and 2.22 U, respectively. Our results correlated with the serum of *O. mykiss* fed with *Lactobacillus rhamnosus* [71] and the mucus of *O. niloticus* fed a diet containing probiotics [72]. In addition, higher alkaline phosphatase activity was observed at 10 mg/g of the EPS-ZnO NPs diet (0.421 U), which correlated with those fed with EPS (0.423 U) at day 30. Conversely, lower activity was seen with 2 mg/g of EPS (0.255 U) on day 30. In contrast, *Cyprinus carpio* fed with leaf extract of *Euphorbia hirta* revealed a significant enhancement in the ALP compared to a control [73], and ALP was also increased intensely in *L. rohita* fed with 0.01–0.05% *Achyranthes aspera* [74]. In the serum antiprotease assay, fish fed with EPS-ZnO NPs showed elevated levels of 0.270, 0.382, and 0.423 U, compared to those fed with EPS, which showed 0.255, 0.334, and 0.421 U at day 30. However, both diets showed intensified serum antiprotease action, and a significant difference was not noticeable in the control. In line with other studies in fish, enhanced protease and antiprotease activities have been reported following probiotic feeding [75,76].

For an antioxidant assessment, in the current study, *O. mossambicus* fed with the EPS and EPS-ZnO NPs diet showed pointedly elevated SOD, CAT, and GPx with the experimental diet (*p <* 0.05) compared to the control. A study by Harikrishnan et al. [77] supports these results; enhanced SOD and GPx activity were seen in *E. bruneus* fed with a *P. linteus* diet.

On the other hand, pathogen challenge tests are appropriate to examine the perceptible effect of EPS and EPS-ZnO NPs on the defense tolerance against pathogens. After being exposed to *A. hydrophila* and *V. parahaemolyticus*, fish fed with the EPS and EPS-ZnO NPs diet survived substantially more than the control. Compared to EPS (76% in *A. hydrophila* and 79% in *V. parahaemolyticus*), EPS-ZnO NP-fed fish had a higher survival rate (93% in *A. hydrophila* and 85% in *V. parahaemolyticus*). In agreement with our observations, similar observations were described with Nile tilapia fed a probiotics diet and exposed to *S. agalactiae* [78]; goldfish fed with *E. acetylicum* S01 and exposed to *A. hydrophila* [79]; *O. mossambicus* fed with *B. licheniformis* and exposed to *A. hydrophila* [18]; *L. plantarum* fed a VSG3 diet and exposed to *A. hydrophila* [80]; *L. rohita* fed with VSG2 and exposed to *P. aeruginosa* [81]; and *O. mykiss* fed with combined inactivated *E. faecilis* and mannon oligosaccharides and exposed to *A. salmonicida* [82].

## 5. Conclusions

This study concludes that EPS and EPS-ZnO NPs diets can invoke an immune response, enrich growth, and increase the disease resistance of *O. mossambicus* against *A. hydrophila* and *V. parahaemolyticus* infection. Furthermore, *O. mossambicus* fed with this diet had the highest post-challenge survival rate, indicating an immunostimulant impact. Hence, our evidence suggests that EPS and EPS-ZnO NPs might be good diet supplements for aquaculture environments. This study also paves the way for finding solutions to microbial diseases in aquatic environments, and the diets described here might be a way for aquafarmers to increase the production rate of cultured fish. The study provides more perspectives in the areas of nanotechnology, aquaculture, and immunology.

## Figures and Tables

**Table 1:** Growth performance of *O. mossambicus* fed with the experimental diet (EPS and EPS-ZnO NPs) after 30 days of feeding trial. Different letters correspond to the statistical difference between treated groups.

Parameters	30 Days						
	Control	EPS-2 mg/g	EPS-5 mg/g	EPS-10 mg/g	EPS-ZnO NPs-2 mg/g	EPS-ZnO NPs-5 mg/g	EPS-ZnO NPs-10 mg/g
Initial weight (g)	7.69 ± 1.2 ^a^	7.72 ± 1.4 ^a^	7.70 ± 1.1 ^a^	7.75 ± 1.8 ^a^	7.68 ± 1.4 ^a^	7.73 ± 1.3 ^a^	7.67 ± 1.9 ^a^
Final weight (g)	17.50 ± 0.9 ^b^	22.73 ± 0.2 ^bc^	25.41 ± 0.4 ^bc^	27.25 ± 0.1 ^cd^	24.61 ± 0.3 ^cd^	26.80 ± 0.5 ^cd^	34.42 ± 1.8 ^d^
Specific growth rate (SGR)	1.56 ± 0.3 ^a^	2.12 ± 0.6 ^bc^	2.30 ± 0.2 ^bc^	2.40 ± 0.6 ^cd^	2.23 ± 0.5 ^bc^	2.36 ± 0.4 ^bc^	2.57 ± 0.8 ^d^
Feed conversion ratio (FCR)	1.32 ± 0.2 ^a^	1.22 ± 0.1 ^ab^	1.18 ± 0.3 ^ab^	1.03 ± 0.4 ^bc^	1.08 ± 0.3 ^bc^	0.93 ± 0.2 ^cd^	0.76 ± 0.1 ^d^

## Data Availability

Not applicable.
